# Identifying Alzheimer's Disease by combining plasma biomarkers and cross‐cultural cognitive tests in an ethnically diverse mixed memory clinic cohort

**DOI:** 10.1002/alz70857_096671

**Published:** 2025-12-24

**Authors:** Daniel Kjaergaard

**Affiliations:** ^1^ Danish Dementia Research Centre, Copenhagen, Copenhagen, Denmark

## Abstract

**Background:**

The need for scalable and accessible measures to identify Alzheimer's Disease (AD) among culturally and linguistically diverse groups is more pressing than ever, especially with growing multiethnic populations worldwide. This need is addressed by cross‐cultural cognitive tests, and by novel plasma biomarkers of AD. However, the added value of combining the two has never been examined. To address this knowledge gap, we conducted a study evaluating the added value of plasma phosphorylated tau 217 (*p*‐tau217) in combination with cross‐cultural cognitive tests in identifying clinical AD among culturally and linguistically diverse patients.

**Method:**

We retrospectively included 337 mixed memory clinic patients across two memory clinics. All patients were born outside the country of examination and were assessed using the cross‐cultural cognitive tests Rowland Universal Dementia Assessment Scale (RUDAS) or the Multicultural Cognitive Examination (MCE). Plasma was analyzed for *p*‐tau217 on a Mesoscale Discovery (MSD) platform. We used k‐fold logistic regression models to determine if combining plasma *p*‐tau217 with cross‐cultural cognitive tests improved the diagnostic accuracy of clinical AD.

**Result:**

The cohort consisted of 89 patients with clinical AD, 138 with other neurological disorders, and 110 neurologically intact patients. Of these, 206 patients had available data on cerebrospinal fluid (CSF) or amyloid‐PET. We found that the cross‐cultural cognitive tests alone accurately identified clinical AD with the RUDAS (AUC: 0.811, SD: 0.100) and MCE (AUC: 0.815, SD: 0.076). Adding *p*‐tau217 significantly increased the diagnostic accuracy for both RUDAS (AUC: 0.909, SD: 0.033) and MCE (AUC: 0.915, SD: 0.056).

**Conclusion:**

We found that supplementing plasma *p*‐tau217 to the RUDAS and MCE significantly improved the diagnostic accuracy of clinical AD in an ethnically diverse mixed memory clinic cohort. These results support the use of culturally appropriate cognitive tests when assessing patients with minority ethnic backgrounds. Furthermore, it promotes combining the cognitive tests with novel plasma biomarkers to aid the diagnosis of AD. Future research should continue to explore other novel blood‐based biomarkers within groups with known barriers in their diagnostic evaluations such as ethnic minorities.

